# Supplementary material for: Antibody-guided in vivo imaging of Aspergillus fumigatus lung infections during antifungal azole treatment
Source: Nat Commun. 2021 Mar 17;12:1707. doi: 10.1038/s41467-021-21965-z (PMC7969596; doi:10.1038/s41467-021-21965-z)
Supplement: Supplementary file 1 — Supplementary Information [file 41467_2021_21965_MOESM1_ESM.pdf]

Antibody-guided in vivo imaging of *Aspergillus fumigatus* lung infections during anti-fungal azole treatment

Supplementary information

Authors:

Sophie Henneberg[1], Anja Hasenberg[1], Andreas Maurer[2], Franziska Neumann[1], Lea Bornemann[1], Irene Gonzalez-Menendez[3], Andreas Kraus[1], Mike Hasenberg[1], Christopher R. Thornton[4], Bernd J. Pichler[2], Matthias Gunzer[1,5*] & Nicolas Beziere[2*].

*corresponding authors: nicolas.beziere@med.uni-tuebingen.de; matthias.gunzer@uni-due.de

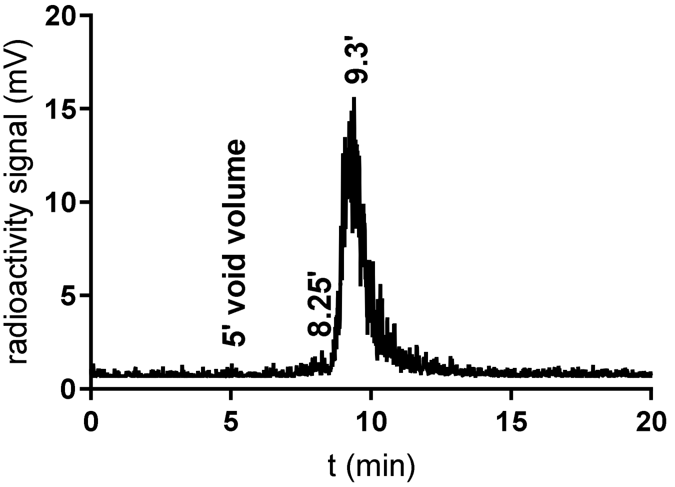

Supplementary Fig. 1 Radio-High Pressure Size Exclusion Chromatography chromatogram of the dual-labeled hJF5-NODAGA-DyLight650.

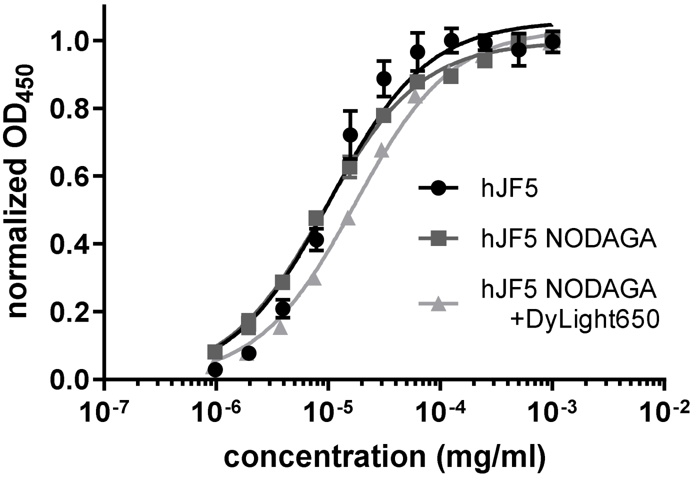

Supplementary Fig. 2 Binding affinity of native hJF5 and its NODAGA or NODAGA and DyLight650 conjugated counterparts. The data shows that there were no significant deleterious effects of the conjugations on the binding affinity of hJF5 to its target antigen in ELISA. *n* = 3 for hJF5, *n* = 1 for hJF5-NODAGA and hJF5-NODAGA-DyLight650. Data are presented as mean values +/- SD where applicable.

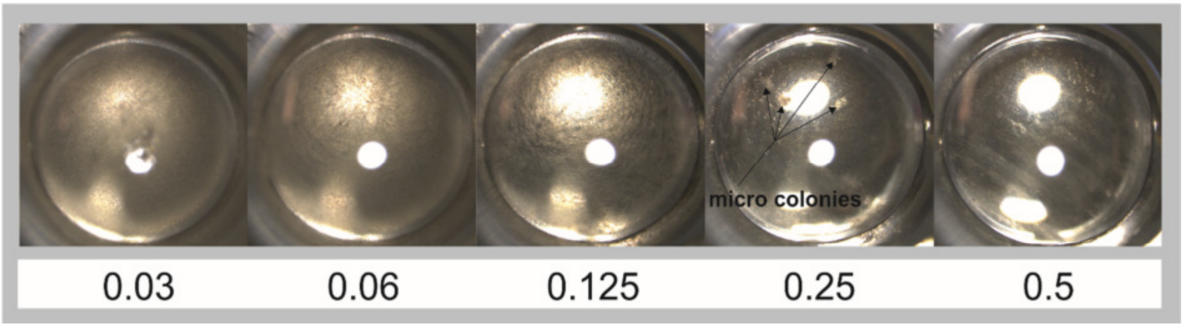

Supplementary Fig. 3 Visual voriconazole susceptibility testing of *A. fumigatus* following EUCAST guidelines. Individual wells of a 96-well flat bottom plate were inoculated with $3x10^4$ conidia of either the *A. fumigatus* wt strain ATCC46645 or the transgenic strain *A. fumigatus*^{tdTomato}. After 48 h the minimum inhibitory concentration (MIC) was determined as voriconazole concentration in the first well of the testing row in which there was no growth detectable. Shown are representative examples of the *A. fumigatus*^{tdTomato} testing. Numbers below the pictures indicate the respective voriconazole concentration. At a concentration of 0.25 µg/ml small "micro colonies" were still visible at the bottom of the plate. Starting at a concentration of 0.5 µg/ml no fungal growth was noticeable anymore.

	MIC of technical replicate #		
	**1**	**2**	**3**
**ATCC46645 - 1**	1 µg/ml	0.5 µg/ml	0.5 µg/ml
**ATCC46645 - 2**	0.5 µg/ml	0.5 µg/ml	0.5 µg/ml
**A. fum.^{tdTomato} - 1**	0.5 µg/ml	0.5 µg/ml	0.5 µg/ml
**A. fum.^{tdTomato} - 2**	0.5 µg/ml	0.5 µg/ml	0.5 µg/ml

Supplementary Table 1 Raw data of the voriconazole susceptibility of *A. fumigatus* strains visual reading assays. In total 6 testing rows per fungal strain (ATCC46645 and *A. fumigatus*^{tdTomato}), with 2 biological replicates and 3 technical replicates were analyzed. Except for the first technical replicate of the first biological replicate of the wt strain all testing rows resulted in a voriconazole minimum inhibitory concentration of 0.5 µg/ml.

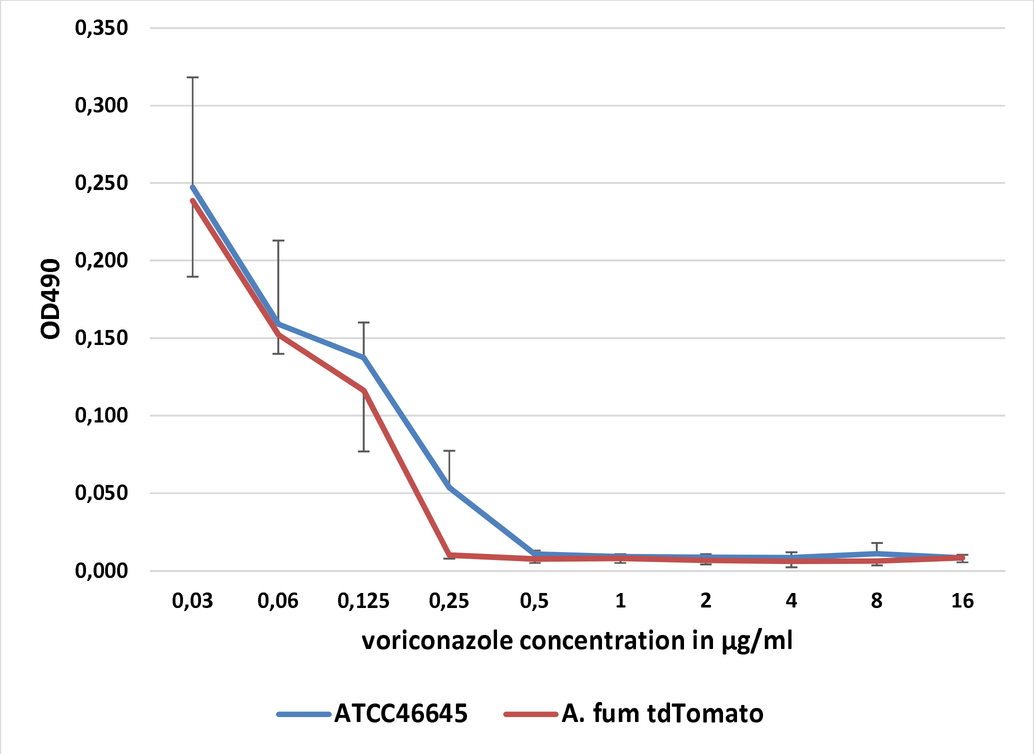

Supplementary Fig. 4 Spectrophotometrical voriconazole susceptibility testing of *A. fumigatus* following EUCAST guidelines. Individual wells of a 96-well flat bottom plate were inoculated with $3x10^4$ conidia of either the *A. fumigatus* wild type strain ATCC46645 or the transgenic strain *A. fumigatus*^{tdTomato}. After 48 h the minimum inhibitory concentration (MIC) was determined by spectrophotometry at 490 nm. In total 6 testing rows per fungal strain were included in the analysis (2 biological replicates, with 3 technical replicates, each.) Data are presented as mean values +/- SD.

Vor. conc [μg/ml]	**0.03**	**0.06**	**0.125**	**0.25**	**0.5**	**1**	**2**	**4**	**8**	**16**
**ATCC46645**	0.247	0.159	0.137	0.054	0.011	0.009	0.009	0.008	0.011	0.008
A. fumigatus$^{\mathbf{tdTomato}}$	0.239	0.152	0.116	0.010	0.008	0.008	0.007	0.006	0.006	0.008

Supplementary Table 2 Mean spectrophotometrical values at 490 nm of the voriconazole susceptibility of *A. fumigatus* strains. Figures are the mean values of 2 biological replicates and 3 technical replicates per fungal strain.

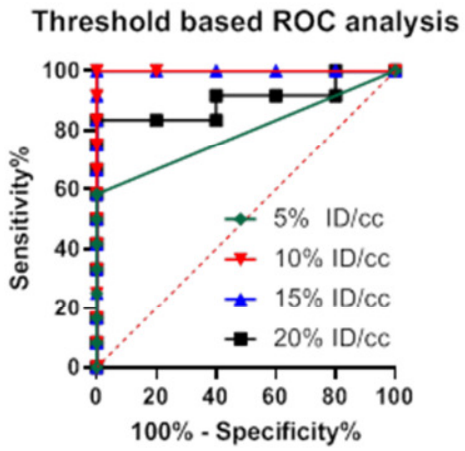

Supplementary Fig. 5 Receiver Operating Characteristic curve. ROC curves for classification between healthy (PBS inoculated, *n* = 5 over 2 independent experiments) and infected (*A. fumigatus* inoculated, *n* = 12 over 3 independent experiments) animals based on various *in vivo* PET signal thresholding values (5, 10, 15 and 20% ID/cc) 48h after injection of ^{64}Cu-hJF5, and 48h after inoculation.

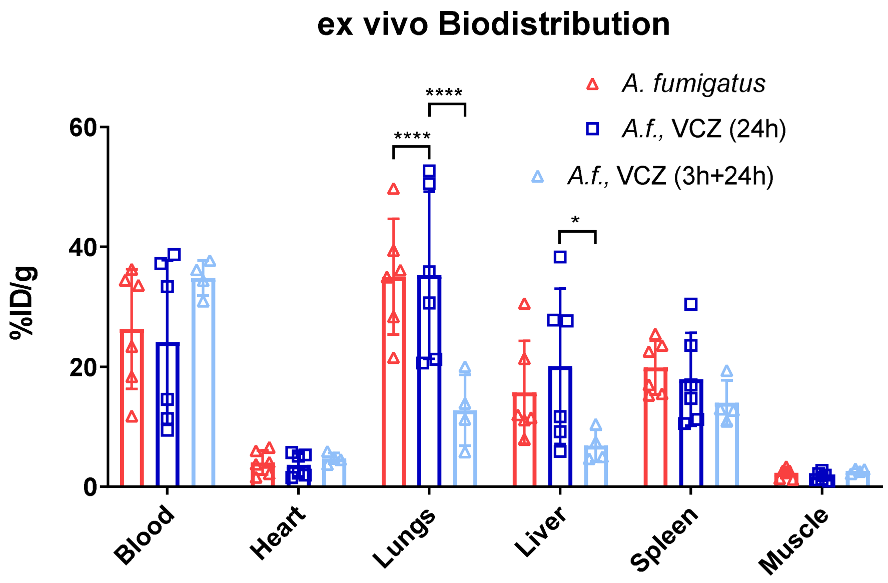

Supplementary Fig. 6 Ex vivo biodistribution data of ^{64}Cu-hJF5. Ex vivo biodistribution data obtained after perfusion of the animals by 0.4% PFA of ^{64}Cu-hJF5 in major organs 48 h after radiotracer injection in *A. fumigatus* infected animals left untreated (*A. fumigatus*, red, n = 6 over 2 independent experiments), receiving voriconazole treatment initiated 24 h after inoculation (*A.f.* VCZ (24h), dark blue, n = 6 over 2 independent experiments), or when initiated at 3 h post-inoculation and at 24 h post-inoculation (*A.f.* VCZ (3+24h), light blue, n = 4). Results are plotted as individual values with average and standard deviation and are expressed as injected dose per gram (ID/g) . All p values were generated using two way ANOVA with Tukey's multiple comparison test (*, $p < 0.05$; **, $p < 0.01$; ***, $p < 0.001$; ****, $p < 0.0001$).

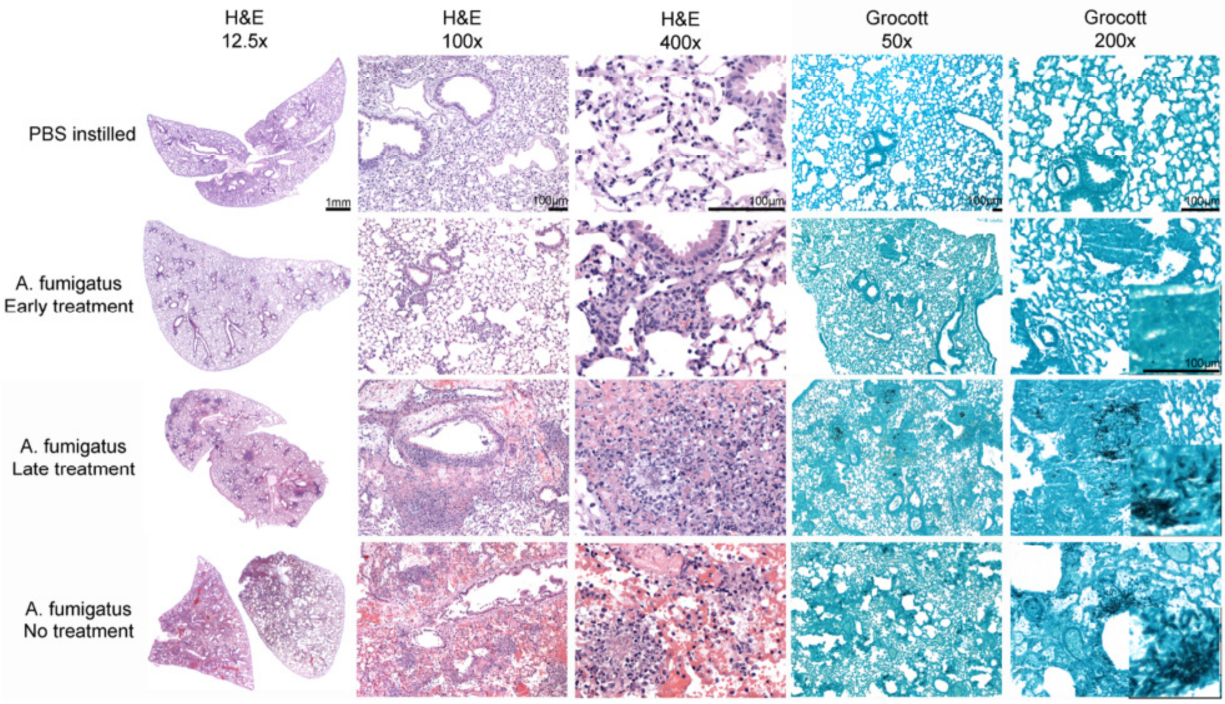

Supplementary Fig. 7 Histological stains of *A. fumigatus* infected lungs during Voriconazole treatment. Hematoxylin and eosin stain (H&E) at 12.5x, 100x and 400x magnification as well as Grocott methenamine silver stain (Grocott) at 50x and 200x magnification of PBS instilled mouse lungs (PBS instilled), *A. fumigatus* infected mice receiving early Voriconazole treatment 3h post infection (*A. fumigatus* Early treatment), *A. fumigatus* instilled mice receiving late Voriconazole treatment 24h post infection (*A. fumigatus* Late treatment) and *A. fumigatus* infected mice not receiving treatment (A. fumigatus No treatment). Images are representative of the entire section for *n* = 3 per group. Of note, one sample in the *A. fumigatus* No treatment group presented a slightly milder phenotype. The scale bars depicted represent the magnification of the entire column and insert where appropriate.

Table of Contents:

Supplementary Fig. 1 Radio-High Pressure Size Exclusion Chromatography chromatogram of the dual-labeled hJF5-NODAGA-DyLight650. 2

Supplementary Fig. 2: Binding affinity of native hJF5 and its NODAGA or NODAGA and DyLight650 conjugated counterparts. 3

Supplementary Fig. 3: Visual voriconazole susceptibility testing of *A. fumigatus* following EUCAST guidelines. 4

Supplementary Table 1: Raw data of the voriconazole susceptibility of *A. fumigatus* strains visual reading assays. 5

Supplementary Fig. 4: Spectrophotometrical voriconazole susceptibility testing of *A. fumigatus* following EUCAST guidelines. 6

Supplementary Table 2: Mean spectrophotometrical values at 490 nm of the voriconazole susceptibility of *A. fumigatus* strains. 7

Supplementary Fig. 5: Receiver Operating Characteristic curve. 8

Supplementary Fig. 6: Ex vivo biodistribution data of ^{64}Cu-hJF5. 9

Supplementary Fig. 7: Histological stains of *A. fumigatus* infected lungs during Voriconazole treatment. 10
