# Supplementary material for: Antibody-guided in vivo imaging of Aspergillus fumigatus lung infections during antifungal azole treatment
Source: Nat Commun. 2021 Mar 17;12:1707. doi: 10.1038/s41467-021-21965-z (PMC7969596; doi:10.1038/s41467-021-21965-z)
Supplement: Supplementary file 3 — Reporting Summary [file 41467_2021_21965_MOESM3_ESM.pdf]

nature research

Corresponding author(s): N. Beziere, M. Gunzer

Last updated by author(s): Jan 14, 2021

Reporting Summary

Nature Research wishes to improve the reproducibility of the work that we publish. This form provides structure for consistency and transparency in reporting. For further information on Nature Research policies, see our Editorial Policies and the Editorial Policy Checklist.

Statistics

For all statistical analyses, confirm that the following items are present in the figure legend, table legend, main text, or Methods section.

n/a	Confirmed	
☐	☒	The exact sample size (n) for each experimental group/condition, given as a discrete number and unit of measurement
☐	☒	A statement on whether measurements were taken from distinct samples or whether the same sample was measured repeatedly
☐	☒	The statistical test(s) used AND whether they are one- or two-sided<br>*Only common tests should be described solely by name; describe more complex techniques in the Methods section.*
☐	☒	A description of all covariates tested
☐	☒	A description of any assumptions or corrections, such as tests of normality and adjustment for multiple comparisons
☐	☒	A full description of the statistical parameters including central tendency (e.g. means) or other basic estimates (e.g. regression coefficient) AND variation (e.g. standard deviation) or associated estimates of uncertainty (e.g. confidence intervals)
☐	☒	For null hypothesis testing, the test statistic (e.g. F, t, r) with confidence intervals, effect sizes, degrees of freedom and P value noted<br>*Give P values as exact values whenever suitable.*
☒	☐	For Bayesian analysis, information on the choice of priors and Markov chain Monte Carlo settings
☒	☐	For hierarchical and complex designs, identification of the appropriate level for tests and full reporting of outcomes
☐	☒	Estimates of effect sizes (e.g. Cohen's d, Pearson's r), indicating how they were calculated

Our web collection on statistics for biologists contains articles on many of the points above.

Software and code

Policy information about availability of computer code

Data collection	Data collection was performed using commercial softwares described in the Methods section: GraphPad Prism 9.0.0, ImSpector, Leica Application Suite X, ImarisFileConverterx64 9.4.X and 9.5.X, Inveon Acquisition Workplace v2.1.271 and Paravision v6.
Data analysis	Data analysis was performed using commercial softwares described in the Methods section: GraphPad Prism 9.0.0, Imaris, Inveon Research Workplace v4.2 and Adobe Photoshop CS6.

For manuscripts utilizing custom algorithms or software that are central to the research but not yet described in published literature, software must be made available to editors and reviewers. We strongly encourage code deposition in a community repository (e.g. GitHub). See the Nature Research guidelines for submitting code & software for further information.

Data

Policy information about availability of data

All manuscripts must include a data availability statement. This statement should provide the following information, where applicable:

- Accession codes, unique identifiers, or web links for publicly available datasets
- A list of figures that have associated raw data
- A description of any restrictions on data availability

Due to their large size, the raw imaging data that support the findings of this study are directly available from the corresponding authors upon reasonable request. Derived data have been compiled in the source data file provided with this paper.

Field-specific reporting

Please select the one below that is the best fit for your research. If you are not sure, read the appropriate sections before making your selection.

☒ Life sciences ☐ Behavioural & social sciences ☐ Ecological, evolutionary & environmental sciences

For a reference copy of the document with all sections, see nature.com/documents/nr-reporting-summary-flat.pdf

Life sciences study design

All studies must disclose on these points even when the disclosure is negative.

Sample size	Sample sizes were chosen based on previously published studies using similar conditions (see article: Davies G, et al. Towards Translational ImmunoPET/MR Imaging of Invasive Pulmonary Aspergillosis: The Humanised Monoclonal Antibody JF5 Detects Aspergillus Lung Infections In Vivo. Theranostics 7, 3398-3414 (2017).)
Data exclusions	Infected animals were removed from the study based on previously established criteria of weight loss, which had to be between 5% and 20% between the day of infection and the 48h timepoint, indicating a failed infection or requiring ethical sacrifice respectively.
Replication	Experimental finding of the control groups were cross-validated using previously published results. Other experimental results were confirmed by replication as indicated for each group in the figure legends. All attempts at replication were succesful. Of note, of the 3 A. fumigatus infected animals receiving no treatment used for histology, 1 presented a slightly milder phenotype.
Randomization	For all experiments, mice were then randomly split into groups after a minimum 1 week of resting time after delivery.
Blinding	Immediately after infection, mice were separated by experimental groups to avoid cross contamination, in litters of maximum 6 littermates. Thus, no blinding of the investigator was possible.

Reporting for specific materials, systems and methods

We require information from authors about some types of materials, experimental systems and methods used in many studies. Here, indicate whether each material, system or method listed is relevant to your study. If you are not sure if a list item applies to your research, read the appropriate section before selecting a response.

Materials & experimental systems

n/a	Involved in the study	
☐	☒	Antibodies
☒	☐	Eukaryotic cell lines
☒	☐	Palaeontology and archaeology
☐	☒	Animals and other organisms
☒	☐	Human research participants
☒	☐	Clinical data
☒	☐	Dual use research of concern

Methods

n/a	Involved in the study	
☒	☐	ChIP-seq
☒	☐	Flow cytometry
☒	☐	MRI-based neuroimaging

Antibodies

Antibodies used	1. hJF5 produced by C. Thornton's team, conugated to NODAGA at 3mg/mL and conjugated to NODAGA and DyLight650 at 1.25 mg/mL<br>2. anti-Ly6G/anti-Ly6c(Gr-1) antibody (#BE0075, BioXCell) diluted in PBS to 1 mg/mL
Validation	1. See publication "Davies G, et al. Towards Translational ImmunoPET/MR Imaging of Invasive Pulmonary Aspergillosis: The Humanised Monoclonal Antibody JF5 Detects Aspergillus Lung Infections In Vivo. Theranostics 7, 3398-3414 (2017)" for complete antibody production and characterisation.<br>2. Commercial antibody, clone RB6-8C5, depleting neutrophils: Conlan, J. W., and R. J. North. 1994. J. Exp. Med.179:259-268 ; Czuprynski, C. J., J. F. Brown, N. Maroushek, R. D. Wagner, and H. Steinberg. 1994. J. Immunol.152:1836-1846.

Animals and other organisms

Policy information about studies involving animals; ARRIVE guidelines recommended for reporting animal research

Laboratory animals	For all experiments, C57BL/6JOlaHsd mice were used. Eight to ten-week-old female mice were purchased from Envigo (Huntingdon, Cambridgeshire, UK), and kept in a standardized and sterile environment (20 ± 1 °C room temperature, 50 ± 10 % relative humidity, 12 h light-dark cycle), with food and water provided ad libitum.

Wild animals	No wild animals were used for this study.
Field-collected samples	No field-collected samples were used in this study.
Ethics oversight	All experiments were undertaken in accordance to the animal welfare act and the EU directive 2010/63/EU, the German animal welfare act and after review and approval by the responsible local ethical committees and authoritiesaccording to the German Animal Protection Law, with permission from the local responsible authorities (Regierungspräsidium Tübingen and Essen, Germany).

Note that full information on the approval of the study protocol must also be provided in the manuscript.
